# Diaqua­bis[4-(dimethyl­amino)­benzoato]-κ^2^ *O*,*O*′;κ*O*-(isonicotinamide-κ*N* ^1^)cobalt(II)

**DOI:** 10.1107/S1600536809016602

**Published:** 2009-05-14

**Authors:** Tuncer Hökelek, Hakan Dal, Barış Tercan, Özgür Aybirdi, Hacali Necefoğlu

**Affiliations:** a Department of Physics, Hacettepe University, 06800 Beytepe, Ankara, Turkey; b Department of Chemistry, Faculty of Science, Anadolu University, 26470 Yenibağlar, Eskişehir, Turkey; c Department of Physics, Karabük University, 78050 Karabük, Turkey; d Department of Chemistry, Kafkas University, 63100 Kars, Turkey

## Abstract

The title Co^II^ complex, [Co(C_9_H_10_NO_2_)_2_(C_6_H_6_N_2_O)(H_2_O)_2_], contains two 4-dimethyl­amino­benzoate (DMAB) anions, one isonicotinamide (INA) ligand and two coordinated water mol­ecules. One of the DMAB anions acts as a bidentate ligand, while the other is monodentate. The four O atoms in the equatorial plane around the Co atom form a highly distorted square-planar arrangement, while the distorted octa­hedral coordination geometry is completed by the N atom of the INA ligand and the O atom of the second water mol­ecule in the axial positions. An intra­molecular O—H⋯O hydrogen bond between the monodentate-coordinated carboxyl group and a coordinated water mol­ecule results in a six-membered ring with an envelope conformation. The dihedral angles between the carboxyl groups and the adjacent benzene rings are 4.29 (10)° for the monodentate ligand and 2.31 (13)° for the bidentate ligand, while the two benzene rings are oriented at a dihedral angle of 65.02 (5)°. The dihedral angles between the pyridine and benzene rings are 11.21 (5)° for the monodentate ligand and 74.60 (5)° for the bidentate ligand. In the crystal structure, inter­molecular O—H⋯O, O—H⋯N and N—H⋯O hydrogen bonds link the mol­ecules into a supra­molecular structure.

## Related literature

For general background, see: Adiwidjaja *et al.* (1978 ▶); Amiraslanov *et al.* (1979 ▶); Antolini *et al.* (1982 ▶); Antsyshkina *et al.* (1980 ▶); Bigoli *et al.* (1972 ▶); Catterick *et al.* (1974 ▶); Chen & Chen (2002 ▶); Hauptmann *et al.* (2000 ▶); Krishnamachari (1974 ▶); Shnulin *et al.* (1981 ▶). For related structures, see: Hökelek *et al.* (1995 ▶, 1997 ▶, 2007 ▶, 2008 ▶); Hökelek & Necefoğlu (1996 ▶, 1997 ▶, 2007 ▶).
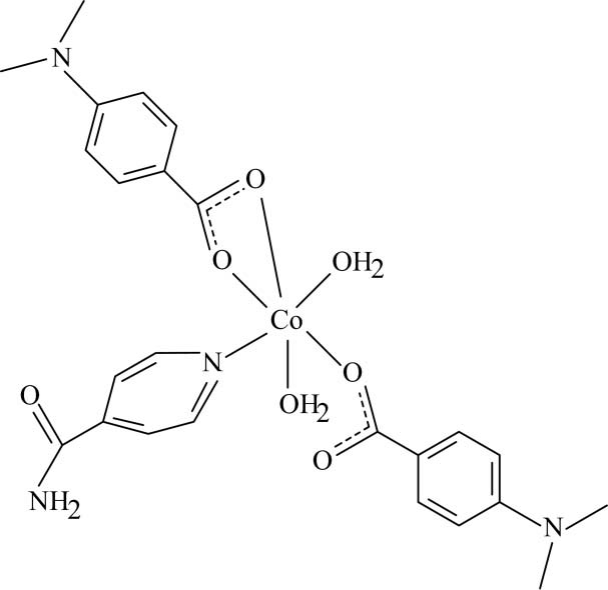


## Experimental

### 

#### Crystal data

[Co(C_9_H_10_NO_2_)_2_(C_6_H_6_N_2_O)(H_2_O)_2_]
*M* *_r_* = 545.45
Triclinic, 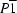

*a* = 6.85550 (10) Å
*b* = 8.1028 (2) Å
*c* = 22.4642 (3) Å
α = 90.9180 (10)°
β = 92.965 (2)°
γ = 93.230 (2)°
*V* = 1243.98 (4) Å^3^
*Z* = 2
Mo *K*α radiation
μ = 0.74 mm^−1^
*T* = 100 K
0.50 × 0.30 × 0.16 mm

#### Data collection

Bruker Kappa APEXII CCD area-detector diffractometer
Absorption correction: multi-scan (*SADABS*; Bruker, 2005 ▶) *T*_min_ = 0.764, *T*_max_ = 0.884
21784 measured reflections
6167 independent reflections
5279 reflections with *I* > 2σ(*I*)
*R*_int_ = 0.046

#### Refinement

*R*[*F* ^2^ > 2σ(*F* ^2^)] = 0.030
*wR*(*F* ^2^) = 0.074
*S* = 1.04
6167 reflections
353 parameters
6 restraints
H atoms treated by a mixture of independent and constrained refinement
Δρ_max_ = 0.43 e Å^−3^
Δρ_min_ = −0.38 e Å^−3^

### 

Data collection: *APEX2* (Bruker, 2007 ▶); cell refinement: *SAINT* (Bruker, 2007 ▶); data reduction: *SAINT*; program(s) used to solve structure: *SHELXS97* (Sheldrick, 2008 ▶); program(s) used to refine structure: *SHELXL97* (Sheldrick, 2008 ▶); molecular graphics: *ORTEP-3 for Windows* (Farrugia, 1997 ▶); software used to prepare material for publication: *WinGX* (Farrugia, 1999 ▶).

## Supplementary Material

Crystal structure: contains datablocks I, global. DOI: 10.1107/S1600536809016602/xu2520sup1.cif

Structure factors: contains datablocks I. DOI: 10.1107/S1600536809016602/xu2520Isup2.hkl

Additional supplementary materials:  crystallographic information; 3D view; checkCIF report

## Figures and Tables

**Table 1:** Selected bond lengths (Å)

Co1—O1	2.0397 (10)
Co1—O3	2.1845 (11)
Co1—O4	2.1445 (11)
Co1—O6	2.0410 (11)
Co1—O7	2.1490 (10)
Co1—N3	2.1314 (12)

**Table 2:** Hydrogen-bond geometry (Å, °)

*D*—H⋯*A*	*D*—H	H⋯*A*	*D*⋯*A*	*D*—H⋯*A*
N4—H41⋯O3^i^	0.857 (18)	2.189 (19)	3.0426 (17)	173.8 (17)
N4—H42⋯O4^ii^	0.88 (2)	1.96 (2)	2.8101 (17)	161.9 (16)
O6—H61⋯N1^iii^	0.918 (17)	1.956 (18)	2.8494 (17)	163.9 (17)
O6—H62⋯O2^iv^	0.90 (2)	1.77 (2)	2.6640 (15)	172 (2)
O7—H71⋯O2	0.914 (15)	1.774 (16)	2.6532 (15)	160.5 (15)
O7—H72⋯O5^v^	0.879 (18)	1.875 (18)	2.7478 (15)	171.6 (17)

Symmetry codes: (i) 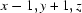; (ii) 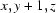; (iii) 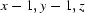; (iv) 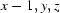; (v) 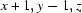.

**Fig. 1.**
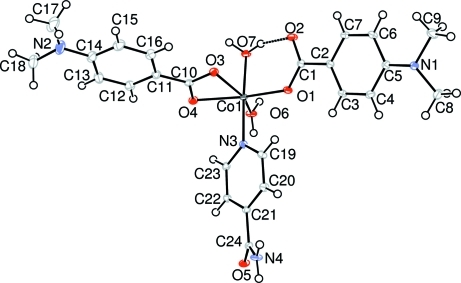
The molecular structure of the title molecule with the atom-numbering scheme. Displacement ellipsoids are drawn at the 50% probability level. Hydrogen bond is shown as dashed line.

## supplementary crystallographic information

### Comment

Nicotinamide (NA) is one form of niacin. A deficiency of this vitamin leads to loss of copper from the body, known as pellagra disease. Victims of pellagra show unusually high serum and urinary copper levels (Krishnamachari, 1974). The nicotinic acid derivative *N*,*N*-diethylnicotinamide (DENA) is an important respiratory stimulant (Bigoli *et al.*, 1972). Transition metal complexes with biochemical molecules show interesting physical and/or chemical properties, through which they may find applications in biological systems (Antolini *et al.*, 1982). Some benzoic acid derivatives, such as 4-aminobenzoic acid, have been extensively reported in coordination chemistry, as bifunctional organic ligands, due to the varieties of their coordination modes (Chen & Chen, 2002; Amiraslanov *et al.*, 1979; Hauptmann *et al.*, 2000).

The structure–function–coordination relationships of the arylcarboxylate ion in Co^II^ complexes of benzoic acid derivatives may also change depending on the nature and position of the substituted groups on the benzene ring, the nature of the additional ligand molecule or solvent, and the pH and temperature of synthesis, as in Zn^II^ complexes (Shnulin *et al.*, 1981; Antsyshkina *et al.*, 1980; Adiwidjaja *et al.*, 1978). When pyridine and its derivatives are used instead of water molecules, the structure is completely different (Catterick *et al.*, 1974).

The structure determination of the title compound, (I), a cobalt complex with two 4-dimethylaminobenzoate (DMAB) and one isonicotinamide (INA) ligands and two water molecules, was undertaken in order to determine the properties of the ligands and also to compare the results obtained with those reported previously.

In the monomeric title complex, (I), the Co atom is surrounded by two DMAB and INA ligands and two water molecules. One of the DMAB ions acts as a bidentate ligand, while the other and INA are monodentate ligands (Fig. 1). The four O atoms (O1, O3, O4 and O6 atoms) in the equatorial plane around the Co atom form a highly distorted square-planar arrangement, while the distorted octahedral coordination is completed by the N atom of the INA ligand (N1) and the O atom of the water molecule (O7) in the axial positions (Table 1 and Fig. 1).

The near equality of the C1—O1 [1.2682 (17) Å], C1—O2 [1.2628 (17) Å], C10—O3 [1.2743 (18) Å] and C10—O4 [1.2716 (18) Å] bonds in the carboxylate group indicates a delocalized bonding arrangement, rather than localized single and double bonds, and may be compared with the corresponding distances: 1.256 (6) and 1.245 (6) Å in [Mn(DENA)_2_(C_7_H_4_ClO_2_)_2_(H_2_O)_2_], (II) (Hökelek *et al.*, 2008), 1.265 (6) and 1.275 (6) Å in [Mn(C_9_H_10_NO_2_)_2_(H_2_O)_4_]. 2(H_2_O), (III) (Hökelek & Necefoğlu, 2007), 1.260 (4) and 1.252 (4) Å in [Zn(DENA)_2_(C_7_H_4_FO_2_)_2_(H_2_O)_2_], (IV) (Hökelek *et al.*, 2007), 1.259 (9) and 1.273 (9) Å in Cu_2_(DENA)_2_(C_6_H_5_COO)_4_, (V) (Hökelek *et al.*, 1995), 1.279 (4) and 1.246 (4) Å in [Zn_2_(DENA)_2_(C_7_H_5_O_3_)_4_]. 2H_2_O, (VI) (Hökelek & Necefoğlu, 1996), 1.251 (6) and 1.254 (7) Å in [Co(DENA)_2_(C_7_H_5_O_3_)_2_(H_2_O)_2_], (VII) (Hökelek & Necefoğlu, 1997) and 1.278 (3) and 1.246 (3) Å in [Cu(DENA)_2_(C_7_H_4_NO_4_)_2_(H_2_O)_2_], (VIII) (Hökelek *et al.*, 1997). In (I), the average Co—O bond length is 2.1117 (11) Å and the Co atom is displaced out of the least-squares planes of the carboxylate groups (O1/C1/O2) and (O3/O4/C10) by -0.536 (1) Å and -0.012 (1) Å, respectively. The dihedral angle between the planar carboxylate groups and the adjacent benzene rings A (C2–C7) and B (C11–C16) are 4.29 (10)° and 2.31 (13)°, respectively, while those between rings A, B and C (N3/C19–C23) are A/B = 65.02 (5), A/C = 11.21 (5) and B/C = 74.60 (5)°. Intramolecular C—H···O hydrogen bond (Table 2) results in the formation of a six-membered ring D (Co1/O1/O2/O7/C1/H71) adopting envelope conformation, with atom Co1 displaced by 0.635 (1) Å from the plane of the other ring atoms.

In the crystal structure, strong intermolecular O—H···O, O—H···N and N—H···O hydrogen bonds (Table 2) link the molecules into a supramolecular structure, in which they may be effective in the stabilization of the structure.

### Experimental

The title compound was prepared by the reaction of CoSO_4_.H_2_O (1.40 g, 5 mmol) in H_2_O (30 ml) and INA (1.22 g, 10 mmol) in H_2_O (20 ml) with sodium 4-dimethylaminobenzoate (1.65 g, 10 mmol) in H_2_O (50 ml). The mixture was filtered and set aside to crystallize at ambient temperature for one week, giving brown single crystals.

### Refinement

H atoms of water molecules and NH_2_ group were located in difference Fourier maps and refined isotropically, with restraints of O6—H61 = 0.919 (14), O6—H62 = 0.903 (16), O7—H71 = 0.910 (14), O7—H72 = 0.881 (15) Å and H61—O6—H62 = 105.6 (18) and H71—O7—H72 = 105.6 (18)°. The remaining H atoms were positioned geometrically with C—H = 0.93 and 0.96 Å, for aromatic and methyl H atoms, respectively, and constrained to ride on their parent atoms, with *U*_iso_(H) = *xU*_eq_(C), where *x* = 1.5 for methyl H and *x* = 1.2 for aromatic H atoms.

### Crystal data

**Table:**

[Co(C_9_H_10_NO_2_)_2_(C_6_H_6_N_2_O)(H_2_O)_2_]	*Z* = 2
*M**_r_* = 545.45	*F*(000) = 570
Triclinic, *P*1	*D*_x_ = 1.456 Mg m^−^^3^
Hall symbol: -P 1	Mo *K*α radiation, λ = 0.71073 Å
*a* = 6.8555 (1) Å	Cell parameters from 9963 reflections
*b* = 8.1028 (2) Å	θ = 2.5–28.3°
*c* = 22.4642 (3) Å	µ = 0.74 mm^−^^1^
α = 90.918 (1)°	*T* = 100 K
β = 92.965 (2)°	Block, brown
γ = 93.230 (2)°	0.50 × 0.30 × 0.16 mm
*V* = 1243.98 (4) Å^3^	

### Data collection

**Table:**

Bruker Kappa APEXII CCD area-detector diffractometer	6167 independent reflections
Radiation source: fine-focus sealed tube	5279 reflections with *I* > 2σ(*I*)
graphite	*R*_int_ = 0.046
φ and ω scans	θ_max_ = 28.4°, θ_min_ = 1.8°
Absorption correction: multi-scan (*SADABS*; Bruker, 2005)	*h* = −9→6
*T*_min_ = 0.764, *T*_max_ = 0.884	*k* = −10→10
21784 measured reflections	*l* = −27→29

### Refinement

**Table:**

Refinement on *F*^2^	Primary atom site location: structure-invariant direct methods
Least-squares matrix: full	Secondary atom site location: difference Fourier map
*R*[*F*^2^ > 2σ(*F*^2^)] = 0.030	Hydrogen site location: inferred from neighbouring sites
*wR*(*F*^2^) = 0.074	H atoms treated by a mixture of independent and constrained refinement
*S* = 1.04	*w* = 1/[σ^2^(*F*_o_^2^) + (0.033*P*)^2^ + 0.378*P*] where *P* = (*F*_o_^2^ + 2*F*_c_^2^)/3
6167 reflections	(Δ/σ)_max_ = 0.001
353 parameters	Δρ_max_ = 0.43 e Å^−^^3^
6 restraints	Δρ_min_ = −0.38 e Å^−^^3^

### Special details

**Table:**

Geometry. All e.s.d.'s (except the e.s.d. in the dihedral angle between two l.s. planes) are estimated using the full covariance matrix. The cell e.s.d.'s are taken into account individually in the estimation of e.s.d.'s in distances, angles and torsion angles; correlations between e.s.d.'s in cell parameters are only used when they are defined by crystal symmetry. An approximate (isotropic) treatment of cell e.s.d.'s is used for estimating e.s.d.'s involving l.s. planes.
Refinement. Refinement of *F*^2^ against ALL reflections. The weighted *R*-factor *wR* and goodness of fit *S* are based on *F*^2^, conventional *R*-factors *R* are based on *F*, with *F* set to zero for negative *F*^2^. The threshold expression of *F*^2^ > σ(*F*^2^) is used only for calculating *R*-factors(gt) *etc*. and is not relevant to the choice of reflections for refinement. *R*-factors based on *F*^2^ are statistically about twice as large as those based on *F*, and *R*- factors based on ALL data will be even larger.

### Fractional atomic coordinates and isotropic or equivalent isotropic displacement parameters (Å^2^)

**Table:**

	*x*	*y*	*z*	*U*_iso_*/*U*_eq_	
Co1	0.43320 (3)	0.02268 (2)	0.277708 (9)	0.00947 (6)	
O1	0.60678 (15)	0.17803 (12)	0.33279 (5)	0.0131 (2)	
O2	0.86084 (15)	0.02285 (12)	0.35203 (5)	0.0149 (2)	
O3	0.61788 (15)	0.00803 (12)	0.20163 (5)	0.0130 (2)	
O4	0.31810 (15)	−0.10110 (12)	0.19727 (5)	0.0131 (2)	
O5	−0.30534 (15)	0.58077 (12)	0.23291 (5)	0.0147 (2)	
O6	0.21656 (16)	−0.06931 (13)	0.32895 (5)	0.0148 (2)	
H61	0.240 (3)	−0.141 (2)	0.3594 (8)	0.039 (6)*	
H62	0.100 (3)	−0.028 (3)	0.3359 (10)	0.048 (7)*	
O7	0.57303 (16)	−0.18934 (13)	0.31208 (5)	0.0134 (2)	
H71	0.688 (2)	−0.137 (2)	0.3264 (9)	0.036 (6)*	
H72	0.605 (3)	−0.258 (2)	0.2840 (8)	0.041 (6)*	
N1	1.23506 (18)	0.74180 (15)	0.43479 (6)	0.0133 (3)	
N2	0.5720 (3)	−0.30713 (19)	−0.06317 (7)	0.0288 (4)	
N3	0.25998 (18)	0.22383 (15)	0.25346 (5)	0.0107 (2)	
N4	−0.0640 (2)	0.76078 (16)	0.20689 (6)	0.0151 (3)	
H41	−0.147 (3)	0.836 (2)	0.2044 (8)	0.018 (5)*	
H42	0.060 (3)	0.784 (2)	0.1996 (9)	0.024 (5)*	
C1	0.7817 (2)	0.15986 (18)	0.35163 (6)	0.0112 (3)	
C2	0.8986 (2)	0.31004 (17)	0.37515 (6)	0.0109 (3)	
C3	0.8160 (2)	0.46310 (18)	0.37877 (7)	0.0134 (3)	
H3	0.6851	0.4713	0.3667	0.016*	
C4	0.9243 (2)	0.60278 (18)	0.39981 (7)	0.0145 (3)	
H4	0.8646	0.7027	0.4025	0.017*	
C5	1.1227 (2)	0.59587 (17)	0.41723 (6)	0.0113 (3)	
C6	1.2061 (2)	0.44248 (18)	0.41310 (7)	0.0136 (3)	
H6	1.3376	0.4342	0.4243	0.016*	
C7	1.0954 (2)	0.30329 (18)	0.39263 (7)	0.0129 (3)	
H7	1.1540	0.2027	0.3905	0.015*	
C8	1.1348 (2)	0.86392 (19)	0.46908 (7)	0.0171 (3)	
H8A	1.2225	0.9587	0.4781	0.026*	
H8B	1.0942	0.8159	0.5055	0.026*	
H8C	1.0223	0.8971	0.4461	0.026*	
C9	1.4336 (2)	0.7219 (2)	0.45897 (9)	0.0234 (4)	
H9A	1.4987	0.8288	0.4665	0.035*	
H9B	1.5039	0.6615	0.4308	0.035*	
H9C	1.4289	0.6624	0.4955	0.035*	
C10	0.4778 (2)	−0.07098 (17)	0.17208 (7)	0.0115 (3)	
C11	0.5002 (2)	−0.12862 (18)	0.11039 (7)	0.0142 (3)	
C12	0.3496 (2)	−0.21833 (19)	0.07857 (7)	0.0180 (3)	
H12	0.2317	−0.2404	0.0964	0.022*	
C13	0.3706 (3)	−0.2755 (2)	0.02120 (8)	0.0226 (4)	
H13	0.2663	−0.3337	0.0008	0.027*	
C14	0.5478 (3)	−0.2470 (2)	−0.00686 (7)	0.0212 (4)	
C15	0.7001 (3)	−0.1550 (2)	0.02534 (8)	0.0221 (4)	
H15	0.8189	−0.1333	0.0080	0.027*	
C16	0.6747 (2)	−0.09685 (19)	0.08242 (7)	0.0175 (3)	
H16	0.7766	−0.0350	0.1026	0.021*	
C17	0.7573 (3)	−0.2808 (3)	−0.09026 (9)	0.0377 (5)	
H17A	0.7532	−0.3397	−0.1278	0.057*	
H17B	0.8596	−0.3202	−0.0644	0.057*	
H17C	0.7824	−0.1648	−0.0967	0.057*	
C18	0.4093 (3)	−0.3871 (2)	−0.09781 (8)	0.0355 (5)	
H18A	0.4509	−0.4176	−0.1364	0.053*	
H18B	0.3058	−0.3125	−0.1022	0.053*	
H18C	0.3631	−0.4844	−0.0778	0.053*	
C19	0.3251 (2)	0.38257 (18)	0.25972 (7)	0.0127 (3)	
H19	0.4555	0.4059	0.2721	0.015*	
C20	0.2078 (2)	0.51366 (18)	0.24869 (7)	0.0130 (3)	
H20	0.2596	0.6221	0.2525	0.016*	
C21	0.0118 (2)	0.48028 (17)	0.23182 (6)	0.0103 (3)	
C22	−0.0572 (2)	0.31584 (17)	0.22533 (7)	0.0114 (3)	
H22	−0.1879	0.2891	0.2143	0.014*	
C23	0.0708 (2)	0.19348 (17)	0.23553 (7)	0.0118 (3)	
H23	0.0243	0.0841	0.2297	0.014*	
C24	−0.1314 (2)	0.61288 (17)	0.22363 (7)	0.0117 (3)	

### Atomic displacement parameters (Å^2^)

**Table:**

	*U*^11^	*U*^22^	*U*^33^	*U*^12^	*U*^13^	*U*^23^
Co1	0.00738 (11)	0.00874 (10)	0.01243 (11)	0.00157 (7)	0.00073 (7)	−0.00010 (7)
O1	0.0090 (5)	0.0130 (5)	0.0173 (6)	0.0029 (4)	−0.0020 (4)	−0.0014 (4)
O2	0.0098 (5)	0.0111 (5)	0.0239 (6)	0.0029 (4)	−0.0005 (4)	0.0000 (4)
O3	0.0104 (5)	0.0124 (5)	0.0160 (6)	0.0000 (4)	0.0006 (4)	−0.0011 (4)
O4	0.0103 (5)	0.0139 (5)	0.0152 (6)	0.0006 (4)	0.0024 (4)	−0.0006 (4)
O5	0.0076 (5)	0.0121 (5)	0.0246 (6)	0.0011 (4)	0.0016 (4)	−0.0013 (4)
O6	0.0102 (6)	0.0172 (5)	0.0182 (6)	0.0052 (4)	0.0041 (5)	0.0056 (4)
O7	0.0101 (6)	0.0119 (5)	0.0185 (6)	0.0029 (4)	0.0006 (5)	−0.0002 (4)
N1	0.0110 (6)	0.0120 (6)	0.0166 (7)	0.0018 (5)	−0.0017 (5)	−0.0023 (5)
N2	0.0426 (10)	0.0311 (8)	0.0138 (7)	0.0097 (7)	0.0043 (7)	−0.0034 (6)
N3	0.0099 (6)	0.0116 (6)	0.0106 (6)	0.0007 (5)	0.0014 (5)	0.0004 (5)
N4	0.0078 (7)	0.0108 (6)	0.0270 (8)	0.0029 (5)	0.0003 (6)	0.0032 (5)
C1	0.0111 (7)	0.0132 (7)	0.0095 (7)	0.0016 (6)	0.0014 (6)	0.0006 (5)
C2	0.0106 (7)	0.0130 (7)	0.0094 (7)	0.0010 (5)	0.0013 (6)	0.0011 (5)
C3	0.0092 (7)	0.0160 (7)	0.0151 (8)	0.0033 (6)	−0.0014 (6)	−0.0006 (6)
C4	0.0138 (8)	0.0121 (7)	0.0179 (8)	0.0041 (6)	0.0006 (6)	−0.0008 (6)
C5	0.0126 (7)	0.0122 (7)	0.0092 (7)	0.0004 (6)	0.0020 (6)	0.0012 (5)
C6	0.0100 (7)	0.0155 (7)	0.0154 (8)	0.0026 (6)	−0.0018 (6)	0.0015 (6)
C7	0.0119 (7)	0.0122 (7)	0.0148 (8)	0.0043 (6)	−0.0003 (6)	0.0008 (6)
C8	0.0209 (9)	0.0145 (7)	0.0159 (8)	0.0026 (6)	0.0009 (7)	−0.0032 (6)
C9	0.0155 (8)	0.0168 (8)	0.0368 (11)	0.0015 (6)	−0.0093 (8)	−0.0055 (7)
C10	0.0118 (7)	0.0080 (6)	0.0150 (8)	0.0027 (5)	0.0007 (6)	0.0019 (5)
C11	0.0161 (8)	0.0127 (7)	0.0141 (8)	0.0031 (6)	0.0013 (6)	0.0010 (6)
C12	0.0164 (8)	0.0199 (8)	0.0177 (8)	0.0020 (6)	0.0016 (7)	−0.0001 (6)
C13	0.0263 (10)	0.0228 (8)	0.0182 (9)	0.0021 (7)	−0.0046 (7)	−0.0023 (7)
C14	0.0322 (10)	0.0190 (8)	0.0133 (8)	0.0090 (7)	0.0016 (7)	0.0016 (6)
C15	0.0234 (9)	0.0249 (9)	0.0194 (9)	0.0053 (7)	0.0087 (7)	0.0028 (7)
C16	0.0187 (8)	0.0171 (7)	0.0171 (8)	0.0016 (6)	0.0025 (7)	0.0015 (6)
C17	0.0527 (14)	0.0452 (12)	0.0177 (10)	0.0161 (10)	0.0122 (9)	−0.0011 (8)
C18	0.0521 (14)	0.0385 (11)	0.0167 (9)	0.0174 (10)	−0.0056 (9)	−0.0080 (8)
C19	0.0090 (7)	0.0134 (7)	0.0155 (8)	−0.0002 (6)	−0.0003 (6)	0.0027 (6)
C20	0.0116 (7)	0.0098 (7)	0.0175 (8)	−0.0018 (5)	0.0002 (6)	0.0012 (6)
C21	0.0093 (7)	0.0120 (7)	0.0101 (7)	0.0022 (5)	0.0024 (6)	0.0011 (5)
C22	0.0089 (7)	0.0128 (7)	0.0123 (7)	−0.0002 (5)	0.0005 (6)	−0.0001 (5)
C23	0.0120 (7)	0.0099 (7)	0.0133 (7)	−0.0003 (5)	0.0007 (6)	−0.0008 (5)
C24	0.0116 (7)	0.0103 (7)	0.0132 (7)	0.0015 (5)	−0.0010 (6)	−0.0019 (5)

### Geometric parameters (Å, °)

**Table:**

Co1—O1	2.0397 (10)	C7—C2	1.390 (2)
Co1—O3	2.1845 (11)	C7—C6	1.382 (2)
Co1—O4	2.1445 (11)	C7—H7	0.9300
Co1—O6	2.0410 (11)	C8—H8A	0.9600
Co1—O7	2.1490 (10)	C8—H8B	0.9600
Co1—N3	2.1314 (12)	C8—H8C	0.9600
Co1—C10	2.5187 (15)	C9—H9A	0.9600
O1—C1	1.2682 (17)	C9—H9B	0.9600
O2—C1	1.2628 (17)	C9—H9C	0.9600
O3—C10	1.2743 (18)	C10—C11	1.473 (2)
O4—C10	1.2716 (18)	C11—C12	1.388 (2)
O5—C24	1.2353 (18)	C11—C16	1.393 (2)
O6—H61	0.919 (14)	C12—C13	1.380 (2)
O6—H62	0.903 (16)	C12—H12	0.9300
O7—H71	0.910 (14)	C13—C14	1.407 (2)
O7—H72	0.881 (15)	C13—H13	0.9300
N1—C5	1.4135 (18)	C15—C14	1.409 (3)
N1—C8	1.4668 (19)	C15—H15	0.9300
N1—C9	1.457 (2)	C16—C15	1.382 (2)
N2—C14	1.370 (2)	C16—H16	0.9300
N2—C17	1.444 (3)	C17—H17A	0.9600
N2—C18	1.443 (3)	C17—H17B	0.9600
N3—C19	1.3405 (19)	C17—H17C	0.9600
N3—C23	1.3456 (19)	C18—H18A	0.9600
N4—C24	1.3269 (19)	C18—H18B	0.9600
N4—H41	0.857 (19)	C18—H18C	0.9600
N4—H42	0.89 (2)	C19—H19	0.9300
C1—C2	1.492 (2)	C20—C19	1.386 (2)
C2—C3	1.395 (2)	C20—C21	1.387 (2)
C3—H3	0.9300	C20—H20	0.9300
C4—C3	1.381 (2)	C22—C21	1.392 (2)
C4—C5	1.401 (2)	C22—C23	1.375 (2)
C4—H4	0.9300	C22—H22	0.9300
C6—C5	1.401 (2)	C23—H23	0.9300
C6—H6	0.9300	C24—C21	1.503 (2)
			
O1—Co1—O3	100.06 (4)	C5—C4—H4	119.6
O1—Co1—O4	159.45 (4)	O5—C24—N4	122.77 (14)
O1—Co1—O6	105.47 (5)	O5—C24—C21	119.20 (13)
O1—Co1—O7	91.43 (4)	N4—C24—C21	118.02 (13)
O1—Co1—N3	89.64 (4)	C4—C3—C2	121.51 (14)
O1—Co1—C10	130.10 (5)	C4—C3—H3	119.2
O3—Co1—C10	30.39 (4)	C2—C3—H3	119.2
O4—Co1—O7	94.57 (4)	N1—C8—H8A	109.5
O4—Co1—O3	60.70 (4)	N1—C8—H8B	109.5
O4—Co1—C10	30.31 (4)	H8A—C8—H8B	109.5
O6—Co1—O3	152.10 (4)	N1—C8—H8C	109.5
O6—Co1—O4	94.88 (4)	H8A—C8—H8C	109.5
O6—Co1—O7	81.01 (4)	H8B—C8—H8C	109.5
O6—Co1—N3	90.00 (4)	C19—C20—C21	118.85 (13)
O6—Co1—C10	124.14 (5)	C19—C20—H20	120.6
O7—Co1—O3	87.37 (4)	C21—C20—H20	120.6
O7—Co1—C10	91.22 (4)	C15—C16—C11	121.55 (16)
N3—Co1—O3	101.34 (4)	C15—C16—H16	119.2
N3—Co1—O4	87.53 (4)	C11—C16—H16	119.2
N3—Co1—O7	170.90 (4)	C23—C22—C21	118.95 (14)
N3—Co1—C10	95.00 (5)	C23—C22—H22	120.5
C1—O1—Co1	127.70 (9)	C21—C22—H22	120.5
C10—O3—Co1	89.46 (9)	N3—C19—C20	123.23 (14)
C10—O4—Co1	91.35 (9)	N3—C19—H19	118.4
Co1—O7—H71	98.5 (13)	C20—C19—H19	118.4
Co1—O7—H72	113.3 (14)	C12—C13—C14	120.86 (16)
H71—O7—H72	105.6 (18)	C12—C13—H13	119.6
Co1—O6—H61	122.1 (13)	C14—C13—H13	119.6
Co1—O6—H62	129.3 (14)	N3—C23—C22	123.42 (13)
H61—O6—H62	105.6 (18)	N3—C23—H23	118.3
C19—N3—C23	117.19 (12)	C22—C23—H23	118.3
C19—N3—Co1	123.17 (10)	C20—C21—C22	118.31 (13)
C23—N3—Co1	119.40 (9)	C20—C21—C24	123.09 (13)
C5—N1—C9	116.82 (12)	C22—C21—C24	118.51 (13)
C5—N1—C8	116.04 (12)	C4—C5—C6	117.65 (13)
C9—N1—C8	111.97 (13)	C4—C5—N1	120.25 (13)
C24—N4—H42	123.3 (12)	C6—C5—N1	121.99 (14)
C24—N4—H41	116.2 (12)	C14—N2—C18	120.59 (16)
H42—N4—H41	120.5 (17)	C14—N2—C17	120.27 (17)
O4—C10—O3	118.49 (14)	C18—N2—C17	119.00 (15)
O4—C10—C11	120.43 (13)	C16—C15—C14	120.68 (16)
O3—C10—C11	121.07 (13)	C16—C15—H15	119.7
O4—C10—Co1	58.34 (8)	C14—C15—H15	119.7
O3—C10—Co1	60.14 (8)	N2—C14—C13	121.32 (17)
C11—C10—Co1	178.69 (11)	N2—C14—C15	121.24 (17)
O2—C1—O1	123.86 (13)	C13—C14—C15	117.43 (15)
O2—C1—C2	118.69 (13)	N1—C9—H9A	109.5
O1—C1—C2	117.45 (12)	N1—C9—H9B	109.5
C6—C7—C2	121.54 (14)	H9A—C9—H9B	109.5
C6—C7—H7	119.2	N1—C9—H9C	109.5
C2—C7—H7	119.2	H9A—C9—H9C	109.5
C12—C11—C16	117.82 (15)	H9B—C9—H9C	109.5
C12—C11—C10	121.25 (14)	N2—C17—H17A	109.5
C16—C11—C10	120.92 (14)	N2—C17—H17B	109.5
C7—C2—C3	117.57 (13)	H17A—C17—H17B	109.5
C7—C2—C1	121.09 (13)	N2—C17—H17C	109.5
C3—C2—C1	121.31 (13)	H17A—C17—H17C	109.5
C7—C6—C5	120.88 (14)	H17B—C17—H17C	109.5
C7—C6—H6	119.6	N2—C18—H18A	109.5
C5—C6—H6	119.6	N2—C18—H18B	109.5
C13—C12—C11	121.64 (16)	H18A—C18—H18B	109.5
C13—C12—H12	119.2	N2—C18—H18C	109.5
C11—C12—H12	119.2	H18A—C18—H18C	109.5
C3—C4—C5	120.84 (14)	H18B—C18—H18C	109.5
C3—C4—H4	119.6		
			
O1—Co1—O3—C10	−172.09 (8)	C6—C7—C2—C3	0.2 (2)
O6—Co1—O3—C10	31.83 (13)	C6—C7—C2—C1	178.40 (14)
N3—Co1—O3—C10	−80.42 (8)	O2—C1—C2—C7	5.3 (2)
O4—Co1—O3—C10	0.19 (8)	O1—C1—C2—C7	−175.29 (14)
O7—Co1—O3—C10	96.92 (8)	O2—C1—C2—C3	−176.59 (14)
O1—Co1—O4—C10	21.94 (16)	O1—C1—C2—C3	2.8 (2)
O6—Co1—O4—C10	−165.93 (8)	C2—C7—C6—C5	0.3 (2)
N3—Co1—O4—C10	104.28 (8)	C16—C11—C12—C13	0.3 (2)
O7—Co1—O4—C10	−84.59 (8)	C10—C11—C12—C13	−178.73 (14)
O3—Co1—O4—C10	−0.19 (8)	C5—C4—C3—C2	1.3 (2)
O6—Co1—O1—C1	112.48 (12)	C7—C2—C3—C4	−1.0 (2)
N3—Co1—O1—C1	−157.62 (12)	C1—C2—C3—C4	−179.19 (14)
O4—Co1—O1—C1	−75.66 (17)	C12—C11—C16—C15	−1.3 (2)
O7—Co1—O1—C1	31.42 (12)	C10—C11—C16—C15	177.74 (14)
O3—Co1—O1—C1	−56.16 (12)	C23—N3—C19—C20	0.0 (2)
C10—Co1—O1—C1	−61.38 (13)	Co1—N3—C19—C20	−174.44 (11)
O1—Co1—N3—C19	21.32 (12)	C21—C20—C19—N3	1.8 (2)
O6—Co1—N3—C19	126.79 (12)	C11—C12—C13—C14	1.1 (2)
O4—Co1—N3—C19	−138.32 (12)	C19—N3—C23—C22	−2.0 (2)
O3—Co1—N3—C19	−78.86 (12)	Co1—N3—C23—C22	172.64 (11)
C10—Co1—N3—C19	−108.92 (12)	C21—C22—C23—N3	2.2 (2)
O1—Co1—N3—C23	−152.97 (11)	C19—C20—C21—C22	−1.5 (2)
O6—Co1—N3—C23	−47.50 (11)	C19—C20—C21—C24	174.91 (14)
O4—Co1—N3—C23	47.38 (11)	C23—C22—C21—C20	−0.3 (2)
O3—Co1—N3—C23	106.84 (11)	C23—C22—C21—C24	−176.92 (13)
C10—Co1—N3—C23	76.79 (11)	O5—C24—C21—C20	−150.39 (15)
Co1—O4—C10—O3	0.33 (13)	N4—C24—C21—C20	29.2 (2)
Co1—O4—C10—C11	179.46 (12)	O5—C24—C21—C22	26.1 (2)
Co1—O3—C10—O4	−0.32 (13)	N4—C24—C21—C22	−154.32 (14)
Co1—O3—C10—C11	−179.45 (12)	C3—C4—C5—C6	−0.6 (2)
O1—Co1—C10—O4	−170.13 (7)	C3—C4—C5—N1	175.64 (14)
O6—Co1—C10—O4	17.01 (10)	C7—C6—C5—C4	−0.1 (2)
N3—Co1—C10—O4	−76.38 (8)	C7—C6—C5—N1	−176.35 (14)
O7—Co1—C10—O4	96.97 (8)	C9—N1—C5—C4	172.91 (14)
O3—Co1—C10—O4	179.67 (13)	C8—N1—C5—C4	37.38 (19)
O1—Co1—C10—O3	10.20 (10)	C9—N1—C5—C6	−11.0 (2)
O6—Co1—C10—O3	−162.65 (7)	C8—N1—C5—C6	−146.50 (14)
N3—Co1—C10—O3	103.95 (8)	C11—C16—C15—C14	0.9 (2)
O4—Co1—C10—O3	−179.67 (13)	C18—N2—C14—C13	6.5 (2)
O7—Co1—C10—O3	−82.70 (8)	C17—N2—C14—C13	−177.97 (16)
Co1—O1—C1—O2	−19.4 (2)	C18—N2—C14—C15	−173.96 (16)
Co1—O1—C1—C2	161.23 (10)	C17—N2—C14—C15	1.6 (2)
O4—C10—C11—C12	−0.8 (2)	C12—C13—C14—N2	178.18 (15)
O3—C10—C11—C12	178.35 (13)	C12—C13—C14—C15	−1.4 (2)
O4—C10—C11—C16	−179.76 (13)	C16—C15—C14—N2	−179.16 (15)
O3—C10—C11—C16	−0.6 (2)	C16—C15—C14—C13	0.4 (2)

### Hydrogen-bond geometry (Å, °)

**Table:**

*D*—H···*A*	*D*—H	H···*A*	*D*···*A*	*D*—H···*A*
N4—H41···O3^i^	0.857 (18)	2.189 (19)	3.0426 (17)	173.8 (17)
N4—H42···O4^ii^	0.88 (2)	1.96 (2)	2.8101 (17)	161.9 (16)
O6—H61···N1^iii^	0.92 (2)	1.96 (2)	2.8494 (17)	164 (2)
O6—H62···O2^iv^	0.90 (2)	1.77 (2)	2.6640 (15)	172 (2)
O7—H71···O2	0.91 (2)	1.77 (2)	2.6532 (15)	161 (2)
O7—H72···O5^v^	0.88 (2)	1.88 (2)	2.7478 (15)	172 (2)

Symmetry codes: (i) *x*−1, *y*+1, *z*; (ii) *x*, *y*+1, *z*; (iii) *x*−1, *y*−1, *z*; (iv) *x*−1, *y*, *z*; (v) *x*+1, *y*−1, *z*.
